# Calcium-to-phosphorus releasing ratio affects osteoinductivity and osteoconductivity of calcium phosphate bioceramics in bone tissue engineering

**DOI:** 10.1186/s12938-023-01067-1

**Published:** 2023-02-09

**Authors:** Pan Jin, Lei Liu, Lin Cheng, Xichi Chen, Shanshan Xi, Tongmeng Jiang

**Affiliations:** 1 Health Science Center, Yangtze University, Jingzhou, 434023 Hubei China; 2 Key Laboratory of Hainan Trauma and Disaster Rescue, The First Affiliated Hospital of Hainan Medical University, Hainan Medical University, Haikou, 571199 China; 3 Key Laboratory of Emergency and Trauma, Ministry of Education, Engineering Research Center for Hainan Bio-Smart Materials and Bio-Medical Devices, Key Laboratory of Hainan Functional Materials and Molecular Imaging, College of Emergency and Trauma, Hainan Medical University, Haikou, 571199 China; 4 Collaborative Innovation Centre of Regenerative Medicine and Medical BioResource Development and Application Co-Constructed By the Province and MinistryGuangxi Medical University, Nanning, 530021 Guangxi China; 5 Articular Surgery, The Second Nanning People’s Hospital, Third Affiliated Hospital of Guangxi Medical University), Nanning, 530031 Guangxi China

**Keywords:** Osteoconductivity, Osteoinductivity, Calcium phosphate, Bioceramics

## Abstract

Calcium phosphate (Ca-P) bioceramics, including hydroxyapatite (HA), biphasic calcium phosphate (BCP), and beta-tricalcium phosphate (β-TCP), have been widely used in bone reconstruction. Many studies have focused on the osteoconductivity or osteoinductivity of Ca-P bioceramics, but the association between osteoconductivity and osteoinductivity is not well understood. In our study, the osteoconductivity of HA, BCP, and β-TCP was investigated based on the osteoblastic differentiation in vitro and in situ as well as calvarial defect repair in vivo, and osteoinductivity was evaluated by using pluripotent mesenchymal stem cells (MSCs) in vitro and heterotopic ossification in muscles in vivo. Our results showed that the cell viability, alkaline phosphatase activity, and expression of osteogenesis-related genes, including osteocalcin (*Ocn*), bone sialoprotein (*Bsp*), alpha-1 type I collagen (*Col1a1*), and runt-related transcription factor 2 (*Runx2*), of osteoblasts each ranked as BCP > β-TCP > HA, but the alkaline phosphatase activity and expression of osteogenic differentiation genes of MSCs each ranked as β-TCP > BCP > HA. Calvarial defect implantation of Ca-P bioceramics ranked as BCP > β-TCP ≥ HA, but intramuscular implantation ranked as β-TCP ≥ BCP > HA in vivo. Further investigation indicated that osteoconductivity and osteoinductivity are affected by the Ca/P ratio surrounding the Ca-P bioceramics. Thus, manipulating the appropriate calcium-to-phosphorus releasing ratio is a critical factor for determining the osteoinductivity of Ca-P bioceramics in bone tissue engineering.

**Supplementary Information:**

The online version contains supplementary material available at 10.1186/s12938-023-01067-1.

## Introduction

Ca-P bioceramics are currently the gold standard bone substitutes in bone reconstruction due in part to their affordable cost, controllable fabricated structure, and favorable biocompatibility [1]. Hydroxyapatite (HA, Ca_10_(PO_4_)_6_(OH)_2_), biphasic calcium phosphates (BCP), and beta-tricalcium phosphate (β-TCP, Ca_3_(PO_4_)_2_) are the three major types of Ca-P materials. The osteoconductivity and osteoinductivity of Ca-P bioceramics have been widely studied.

The osteoconductivity of Ca-P biomaterials, which supports the continued deposition of bone, has been widely applied in the healing of irregular and large bones, such as craniofacial, mandibular, and calvarial defects [2–4]. Ca-P bioceramics adsorb proteins that are beneficial to osteogenesis and provide a three-dimensional framework for preosteoblasts/osteoblasts, leading to new bone formation and mineralized matrix secretion [5]. Meanwhile, ion concentrations have been shown to play an indispensable role in the ossification process [6, 7]. In a previous study, when BCP and HA were implanted in femoral cortical bone, significantly more bone was found in BCP than in HA [8]. Similarly, in a comparative study on the healing effect of bioceramics on 8-mm-calvarial defect models of rabbits, BCP (HA/β-TCP = 60/40) and BCP (HA/β-TCP = 20/80) exhibited similar osteoconductivity and biodegradation patterns, and both were notably better than pure β-TCP [9]. In addition, in a study considering the implantation of 10 Ca-P materials in the posterior lumbar spine of goat, the osteoconductivity of BCP and β-TCP was demonstrated to be higher than that of HA, and high sintering temperature was found to distinctly impair osteoconductivity [10]. According to these in vivo studies, osteoconductivity is in the order of BCP > β-TCP > HA or BCP ≈ β-TCP > HA. However, Yuan et al. reported that the osteoconductivity of β-TCP is higher than that of BCP and HA after implantation in posterolateral spinal fusion of dogs and illium defect in sheep [11].

Osteoinductivity is the ability to stimulate immature cells into a bone-forming cell lineage and direct bone deposition; in other words, osteogenesis is induced by bone-like tissues in nonossified tissues, such as muscles and tendons, in an ectopic environment [5]. Studies exploring the osteoinductivity of bone alternatives have extensively used mesenchymal stem cells (MSCs) in vitro and ectopic implantation in muscles or subcutaneous sites in vivo [11, 12]. Because of their multidirectional differentiation potential, MSCs are recognized as a promising cell source for cell-based therapy [13–15]. Previous investigations suggested that Ca-P bioceramics trigger osteogenic differentiation of MSCs without the addition of any extraneous growth factors or cytokines [12]. Several in vivo investigations demonstrated new osteoid and bone formation caused by Ca-P bioceramics in the muscles of animals, including baboon, goat, dog, rabbit, rat, and mouse [12, 16, 17]. Enrichment of proteins and growth factors from abundant capillaries was found in Ca-P bioceramics, and these changes were shown to create a microenvironment mimicking the osseous site to direct osteogenic differentiation [18]. Investigating the performance of different scaffolds revealed that TCP induced significantly more bone neoformation in vivo and higher expression of osteogenic differentiation markers in vitro than HA [19], and BCP composed of HA and β-TCP showed stronger osteoinductivity than pure HA or β-TCP [8, 20, 21]. The osteoinductivity of these materials can be ranked as BCP > β-TCP > HA [12]. However, other research reported that the osteoinductivity of TCP is higher than that of BCP and HA in an in vitro model with human multipotent marrow stromal cells (hMSCs) cultured on four types of Ca-P ceramics and in an in vivo study with Ca-P ceramics implanted in sheep intramuscularly [11].

Thus, there is no consensus on the osteoconductivity and osteoinductivity of HA, BCP, and β-TCP in vitro or in vivo, and the association between osteoconductivity and osteoinductivity is not well understood. More research is needed to determine the causes of conflicting results among previous studies. In our study, HA, BCP, and β-TCP were adopted for in situ bone repair and ectopic bone formation to investigate the link between osteoconductivity and osteoinductivity. HA1250 (HA sintered at 1250 °C), which possesses minor bioactivity because of its high sintering temperature [1], was used as the control material. This study may provide new insights into the rational design of Ca-P bioceramics in bone tissue engineering.

## Materials and methods

### Material preparation and characterization

Ca-P powder was synthesized by a wet chemical method at the National Engineering Research Center for Biomaterials of Sichuan University, China. BCP was composed of 60% HA and 40% β-TCP. Samples were machined into *Φ*12 mm × 2 mm plates and *Φ*5 mm × 2 mm plates. HA, BCP, and β-TCP porous bioceramics were fabricated by a hydrogen peroxide (H_2_O_2_) forming method as previously described [22], and HA1250 plates were set as the control. All bioceramics had a similar interconnected pore structure with a porosity of 75 ± 5%. X-ray diffraction (XRD) was performed using Empyrean (Netherlands) and further analyzed using MDI Jade 6. Microstructures were examined with a scanning electron microscope (SEM, Regulus 8100, HITACHI company, Tokyo, Japan).

### In vitro experiment

#### Cell culture

Primary osteoblasts were harvested from the bilateral parietal bone of 5-day-old Sprague–Dawley (SD) rats by enzymatic digestion, and bone marrow stem cells (BMSCs) were collected from the bone marrow extract by the adherent culture method (Animal Resources Centre of Guangxi Medical University, Nanning, Guangxi, China). After being stripped clearly with sterile gauze and digested with 0.25% trypsin−ethylene diamine tetraacetic acid (EDTA) (Beijing Solarbio Science and Technology Co., Ltd., China), the bilateral parietal bones were cut into pieces and digested with 1 mg/mL collagenase type I (Gibco BRL Co., Ltd., Gaithersburg, Maryland, USA) for 3 h. Meanwhile, bone marrow was flushed out of the femoral bone with culture medium. After centrifugation, isolated cells were suspended in alpha-modified Eagle’s medium (α-MEM, Gibco BRL Co., Ltd.) supplemented with 10% (v/v) fetal bovine serum (FBS, Zhejiang Tianhang Biotechnology Co., Ltd., Huzhou, Zhejiang, China) and 1% (v/v) antibiotics and cultured in a 5% CO_2_ incubator (Thermo Scientific TM Forma Series II Water-Jacketed, Santa Ana, California, USA) at 37 °C, with the medium changed every other day. At 80‒90% confluence after about 7 days of culture, primary cells were prepared for subsequent experiments.

#### Cell seeding

Before cell culture, porous scaffolds were sterilized with the alcohol sterilization method described by Li et al. [23]. Immediately before cell seeding, the scaffolds were soaked in α-MEM for 24 h. Primary osteoblasts and BMSCs were suspended in culture medium. Fifty thousand cells diluted into 100 μL culture medium were added to each *Φ*12 mm × 2 mm scaffold and maintained in a 5% CO_2_ humidified incubator at 37 °C. After 100 μL of the culture medium was dropped on the surface of each scaffold per hour four times, 1 mL of the culture medium was slowly added along the sidewall of a 24-well plate to prevent the washing off the unbound cells.

#### Cell proliferation assay

Cell proliferation was analyzed using the 3-(4,5-dimethylthiazol-2-yl)-2,5- diphenyltetrazolium bromide (MTT, Sigma-Aldrich, Saint Louis, Missouri, USA) mitochondrial reaction, which is based on the ability of live cells to reduce the tetrazulium-based compound MTT to a purplish formazan product. Cell viability is proportional to the amount of dehydrogenase activity within the cells. After 7 and 14 days, MTT was added to each well with a final concentration of 0.5 mg/mL. After 4 h of incubation, the supernatant was discarded and 1 mL dimethylsulfoxide (DMSO, Sigma-Aldrich) was added for formazan-crystal solubilization. After continuous gentle shaking for 10 min in the dark to dissolve the crystal thoroughly and evenly, 200 μL of the samples was randomly extracted from each of three parallel wells at the same concentration three times and transferred to 96-well plates. The absorbance value was measured at 570 nm with a microplate reader (Thermo Scientific Multiskan GO Microplate Spectrophotometer, Helsinki, Finland), and the results were recorded as the optical density value.

#### Cell viability assay

Cell viability was assessed using fluorescein diacetate-propidium iodide (FDA-PI) staining on day 7 (FDA, Life Technologies [AB & Invitrogen], Carlsbad, California, USA; PI, Life Technologies [AB & Invitrogen]). Cells were incubated with FDA and PI in the dark at 37 °C for 5 min. Images of the top of Ca-P bioceramics were captured using an inverted phase-contrast microscope (Olympus, Tokyo, Japan).

#### Alkaline phosphatase (ALP) activity assay

ALP activity assay was performed using an ALP detection reagent kit (Nanjing Jiancheng Bioengineering Research Institute, Nanjing, China) according to the manufacturer’s instructions. Culture medium was collected for ALP activity assay after 7 and 14 days. After being centrifuged at 1000 rpm for 10 min, the supernatant of the medium was collected for assay. Briefly, after adding buffer solution, matrix solution, water bath, and coloring, the absorbance value was measured at 520 nm with a microplate reader. All samples were performed in triplicate.

#### Cell actin cytoskeleton detection

To observe the cell actin cytoskeleton, staining for actin filaments was performed. After being cultured for 14 days, cells were washed with PBS, fixed with 4% paraformaldehyde (Sigma-Aldrich), permeabilized with 0.5% Triton-X (Gibco BRL Co., Ltd.), and incubated with rhodamine phalloidin (Cytoskeleton, Inc., Denver, Colorado, USA) and Hoechst 33258 (Sigma-Aldrich) in the dark. Cell cytoskeleton organizations of cells at the top of Ca-P bioceramics were observed with a laser scanning confocal microscope (Nikon A1, Tokyo, Japan).

#### Real-time polymerase chain reaction (RT-PCR) assay

RT-PCR assay was performed to detect the expression of osteocalcin (*Ocn*), bone sialoprotein (*Bsp*), alpha-1 type I collagen (*Col1a1*), and runt-related transcription factor 2 (*Runx2*). On days 7 and 14, total RNA was extracted with an RNA extraction reagent (G3013, Wuhan Servicebio Technology Co., Ltd., Wuhan, Hu Bei, China), trichloromethane (10,006,818, Sinopharm Chemical Reagent Co., Ltd., Shanghai, China), isopropanol (80,109,218, Sinopharm Chemical Reagent Co., Ltd.), and absolute ethyl alcohol (10,009,218, Sinopharm Chemical Reagent Co., Ltd.). An equal amount of RNA (300 ng) was used as the template and was reverse transcribed into cDNA using a reverse transcription kit (Servicebio®RT First Strand cDNA Synthesis Kit, G3330, Wuhan Servicebio Technology Co., Ltd.), which was then amplified by using an SYBR Green mix kit (2 × SYBR Green qPCR Master Mix [None ROX], G3320, Wuhan Servicebio Technology Co., Ltd.) on a real-time fluorescence quantitative instrument (CFX, Bio-Rad, California, USA). The primers for PCR were designed as shown in Table 1 [24–27]. The dissociation curve of each primer pair was analyzed to confirm the primer specificity. Marker gene expressions were analyzed by the 2^−ΔΔCT^ method using β-actin. Each sample was tested three times for each gene.

**Table 1:** Primers for real-time polymerase chain reaction

Gene name	Forward primer	Reverse primer
*β-Actin*[24]	5ʹ-TGCTATGTTGCCCTAGACTTCG-3ʹ	5ʹ-GTTGGCATAGAGGTCTTTACGG-3ʹ
*Ocn*[24]	5ʹ-TGACAAAGCCTTCATGTCCAA-3ʹ	5ʹ-CTCCAAGTCCATTGTTGAGGTAG-3ʹ
*Bsp*[25]	5ʹ- GCTATGAAGGCTACGAGGGTCAGGATTAT -3ʹ	5ʹ- GGGTATGTTAGGGTGGTTAGCAATGGTGT -3ʹ
*Col1a1*[26]	5ʹ-AGAGGCATAAAGGGTCATCGTG-3ʹ	5ʹ- CAGGTTGCAGCCTTGGTTAGG -3ʹ
*Runx2*[27]	5ʹ-CCCAACTTCCTGTGCTCCGT-3ʹ	5ʹ-AGTGAAACTCTTGCCTCGTCC-3ʹ

*β-Actin*: beta-actin (used as the normalization control), *Ocn*: osteocalcin, *Bsp*: bone sialoprotein, *Col1a1*: alpha-1 type I collagen, *Runx2*: runt-related transcription factor 2

### In vivo experiment

#### Surgical procedure

Forty male SD rats weighing 280 ± 20 g were obtained from the Animal Resources Center of Guangxi Medical University (Nanning, Guangxi, China). The animal experiments were approved by the Animal Care and Experiment Committee of Guangxi Medical University (Protocol Number: 201510004). Animals were housed in a temperature-controlled environment and provided a standard diet and water. After being randomly divided into four groups, namely, control, HA, β-TCP, and BCP groups, with 10 rats in each group, rats were anesthetized with chloral hydrate via intraperitoneal injection and sterilized with iodine as described in a previous study [28]. Critically sized defects (5 mm in diameter) were created in five rats of each group using a circular trephine under infusion of saline solution on the right parietal bone and implanted with HA1250, HA, β-TCP, or BCP porous scaffolds. Ceramic discs were implanted into the thigh muscles of the other five rats in each group.

#### Histological analysis

The entire skulls of calvarial defect rats and intramuscularly implanted ceramics were extracted after 3 months. For histological analysis, the extracted skulls were fixed with paraformaldehyde, decalcified with formic acid, embedded in paraffin, sectioned into 5-μm-thick sections, and stained with hematoxylin and eosin (HE).

#### Immunohistochemical staining

For immunohistochemical staining, the sections were heated with citrate buffer for antigen retrieval, blocked with normal goat serum, and then incubated with rabbit polyclonal antiosteocalcin antibody (OCN, 1:200, GB11233, Wuhan Servicebio Technology Co., Ltd.). After being washed with PBS three times, the sections were incubated with biotinylated goat antirabbit antibody (SP-9000, ZSGB-BIO, Beijing, China) and stained with substrate−chromogen solution 3,3ʹ-diaminobenzidine tetrahydrochloride (DAB). Finally, slides were sealed with neutral resin and observed with an inverted phase-contrast microscope (Olympus, Tokyo, Japan). Three images were selected from each group, and the proportion of the positive area was analyzed and statistically analyzed with a digital pathological and immunohistochemical image analysis software based on the artificial intelligence learning (Aipathwell, Wuhan Servicebio Technology Co., Ltd., Wuhan, Hubei, China).

#### Micro-computed tomography (micro-CT) analysis

Three months after implantation, the rats were sacrificed, and the entire portion of the defective skull was removed. The samples were fixed in 10% formalin solution at room temperature. Micro-CT (Skyscan 1076, Antwerp, Belgium) was performed to observe new bone formation at the defect sites. Each sample was fixed on the object stage, and imaging was performed on the sample for 360° of rotation with an exposure rate of 20 min per frame. Micro-CT images were reconstructed using CTAn (Skyscan) and CTVol (Skyscan) to make three-dimensional images. Bone volume and bone volume/total volume were calculated to evaluate the quantity of new bone.

#### Ion concentration detection

Different bioceramics were immersed in deionized water (the volume to volume ratio was 1:10) and maintained in a sterile humidified incubator at 37 °C with 5% CO_2_. After 7 days, the concentrations of calcium and phosphate ions were detected with Inductively Coupled Plasma Optical Emission Spectrometer (ICP-OES, SPECTRO ARCOS, Germany).

### Statistical analysis

All data obtained were recorded as mean ± standard deviation (SD) and compared using one-way analysis of variance (ANOVA). A least significance difference test (LSD-t) was performed for further evaluation of the data. SPSS software (version 26.0, SPSS Inc., Chicago, Illinois, USA) was used for statistical analysis, and P < 0.05 was considered statistically significant.

## Results

### Calcium phosphate (Ca-P) bioceramics exhibited low cytotoxicity to intrascaffold-cultured osteoblasts and BMSCs

**Fig. 1:**
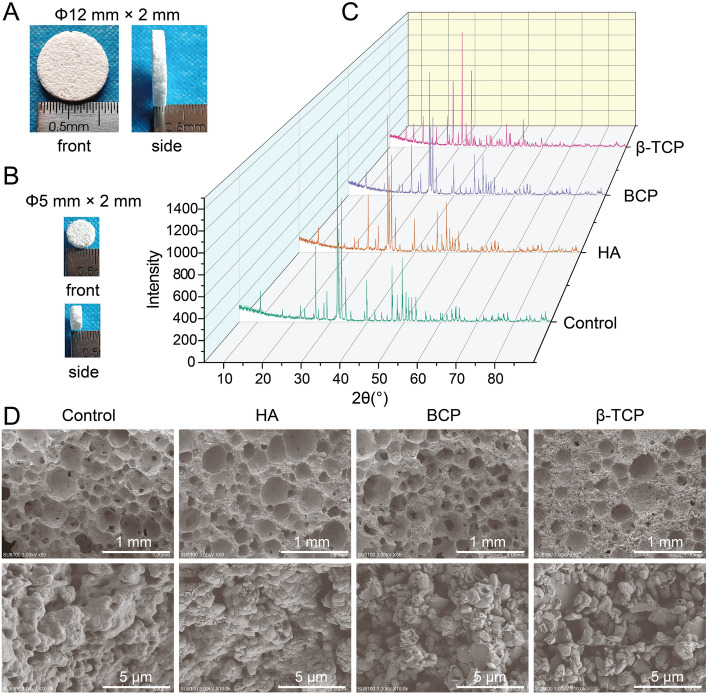
Characteristics of four types of calcium phosphate (Ca-P) bioceramics. **A** Photograph of typical Φ12 mm × 2 mm samples. **B** Photograph of typical Φ5 mm × 2 mm samples. **C** XRD results of four types of Ca-P bioceramics. **D** Typical scanning electron microscope (SEM) photographs of four types of Ca-P bioceramics at 50 × (scale bar = 1 mm) and 10,000 × (scale bar = 5 μm)

**Fig. 2:**
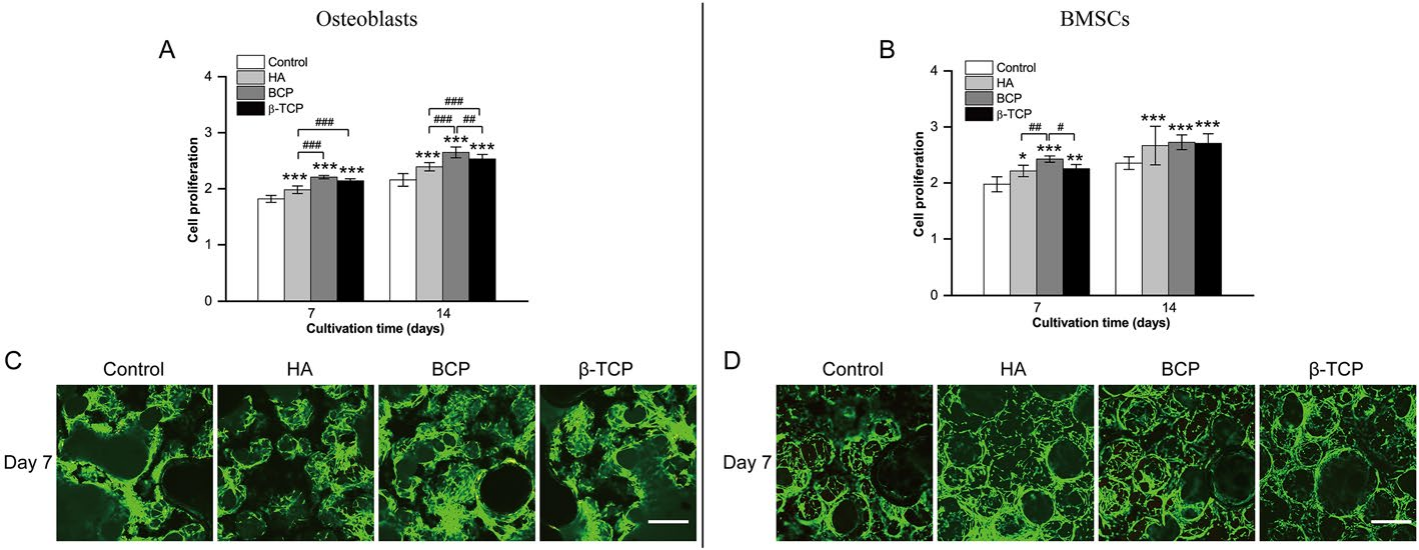
Comparison of cell proliferation and viability of primary osteoblasts and bone marrow stem cells (BMSCs) in the control, HA, BCP, and β-TCP groups. **A**, **B** Cell proliferation of intrascaffold-cultured osteoblasts (**A**) and BMSCs (**B**) in the control, HA, BCP, and β-TCP groups on days 7 and 14 via MTT assay. **C**, **D** Cell viability of intrascaffold-cultured osteoblasts (**C**) and BMSCs (**D**) in the control, HA, BCP, and β-TCP groups on day 7 via live/dead cell assay. All data points are presented as the mean ± standard deviation (*n* = 9). *,# indicates *P* < 0.05; **,## indicates *P* < 0.01; ***,### indicates *P* < 0.001. Live cells were stained green, and dead cells were stained red. Scale bar = 200 μm

Photographs of Φ12 mm × 2 mm and Φ5 mm × 2 mm samples are shown in Fig. 1A, B. The obvious diffraction peaks of the four ceramics were at 2θ = 31.763° (control), 2θ = 31.762° (HA), 2θ = 31.769° (BCP), and 2θ = 31.006° (β-TCP) (Fig. 1C). All the identified formulas in the control (Additional file 1: Table S1), HA (Additional file 2: Table S2), BCP (Additional file 3: Table S3), and β-TCP (Additional file 4: Table S4) groups are listed in the supporting information. To minimize the influence of structural factors on the results of our study, all bioceramic specimens were synthesized with procedure control to keep the pore distribution between 100 and 500 μm (Fig. 1D). Because three-dimensional ex vivo culture closely replicates the structure of cell growth in vivo [29], an intrascaffold-cultured system was adopted to evaluate the cell proliferation and viability of primary osteoblasts and BMSCs. From day 7 to day 14, the numbers of these cells in the same group increased slowly over time (Fig. 2A, B). For osteoblasts or BMSCs in different groups, the numbers of these cells increased faster when they were cultured in HA, BCP, and β-TCP bioceramics than when they were cultured in the control. In addition, more live cells were found in the HA, BCP, and β-TCP bioceramics than in the control (Fig. 2C, D). The viability of BMSCs (Fig. 2D) was consistent with that of osteoblasts (Fig. 2C) on day 7. All these results demonstrated that Ca-P bioceramics have low cytotoxicity and are beneficial to the survival of primary osteoblasts and BMSCs.

### In vitro osteoconductivity of Ca-P bioceramics ranked as BCP > β-TCP > HA, whereas osteoinductivity ranked as β-TCP > BCP > HA

**Fig. 3:**
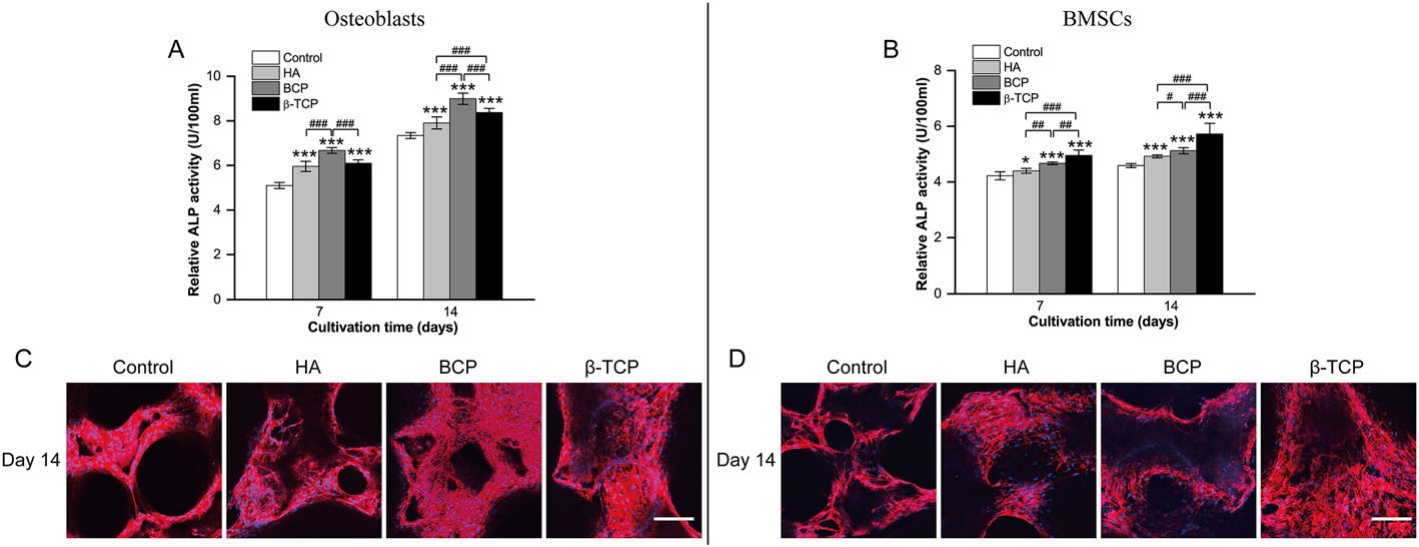
Comparison of osteoconductivity and osteoinductivity of primary osteoblasts and bone marrow stem cells (BMSCs) in the control, HA, BCP, and β-TCP groups in vitro. **A**, **B** Relative alkaline phosphatase (ALP) activity of intrascaffold-cultured osteoblasts (**A**) and BMSCs (**B**) in the control, HA, BCP, and β-TCP groups on days 7 and 14. **C**, **D** Images of rhodamine phalloidin/Hoechst 33,258 staining of intrascaffold-cultured osteoblasts (**C**) and BMSCs (**D**) in the control, HA, BCP, and β-TCP groups on day 14. All data points are presented as the mean ± standard deviation (*n* = 9). *, # indicates *P* < 0.05; **, ## indicates *P* < 0.01; ***, ### indicates *P* < 0.001. Actin cytoskeletons were stained red, and nuclei were stained blue. Scale bar = 100 μm

**Fig. 4:**
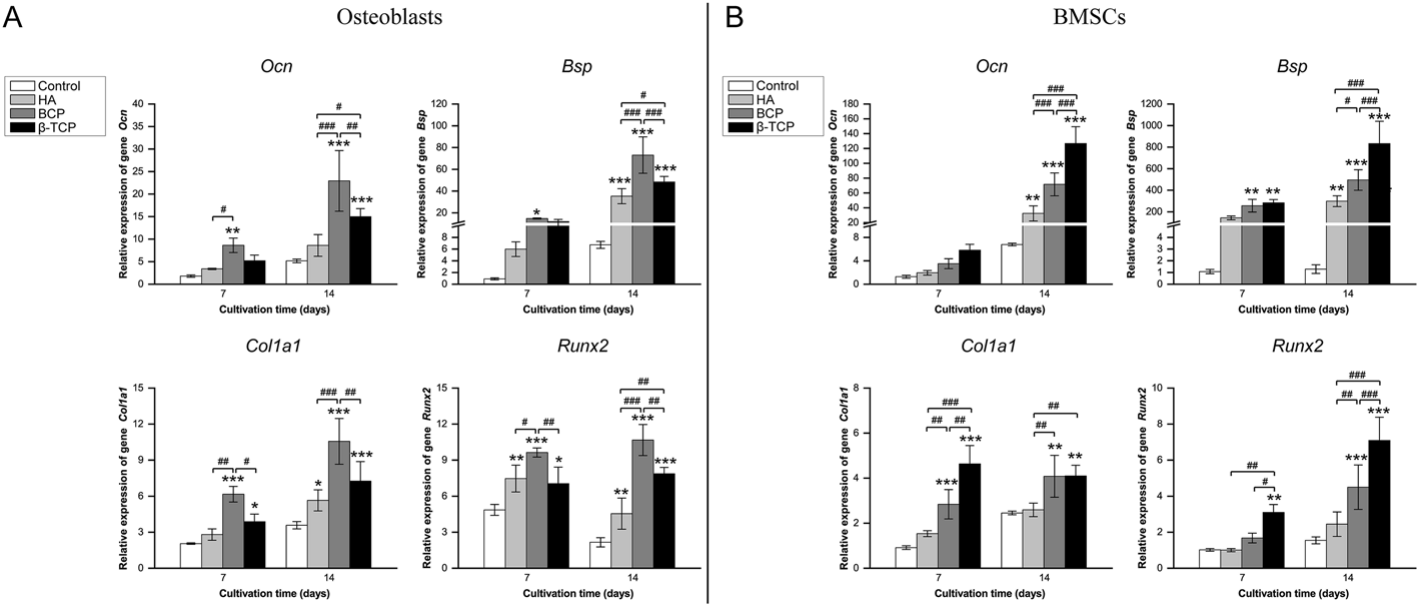
Comparison of osteogenesis-specific genes of primary osteoblasts and bone marrow stem cells (BMSCs) in the control, HA, BCP, and β-TCP groups in vitro. **A**, **B** mRNA expression of osteocalcin (*Ocn*), bone sialoprotein (*Bsp*), alpha-1 type I collagen (*Col1a1*), and runt-related transcription factor 2 (*Runx2*) by primary osteoblasts (**A**) and BMSCs (**B**) in the control, HA, BCP, and β-TCP groups on days 7 and 14. All data points are presented as the mean ± standard deviation (*n* = 3). *, # indicates P < 0.05; **, ## indicates P < 0.01; ***, ### indicates P < 0.001

As composites of HA and β-TCP, BCP scaffolds have been widely used in bone reconstruction [1]. ALP is produced by osteoblasts and is commonly used as a marker of osteogenesis [30]. As shown in Fig. 3A, the ALP activity of osteoblasts cultured in HA, BCP, and β-TCP bioceramics was obviously higher than that of osteoblasts cultured in the control. In Fig. 3B, BMSCs exhibited higher ALP production in the β-TCP group than in the other groups, indicating that porous β-TCP scaffolds may be more beneficial for osteogenesis. Meanwhile, the adherence to scaffolds exhibited by the actin cytoskeletons of osteoblasts and BMSCs was assessed with rhodamine phalloidin/Hoechst 33,258 staining. As shown in Fig. 3C and D, actin filaments were observed in the four groups, indicating that these scaffolds have no significant cytotoxicity. The pro-osteogenic effect of the four Ca-P bioceramics on osteoblasts and BMSCs was further assessed based on the gene expression of *Ocn*, *Bsp*, *Col1a1*, and *Runx2* after 7 and 14 days of culture (Fig. 4). Figure 4A shows that genes closely related with osteogenic differentiation, including *Ocn*, *Bsp*, and *Col1a1*, were all upregulated over time. When compared with the control group, the expressions of osteogenic genes were obviously upregulated in the HA, BCP, and β-TCP groups. Except for slight variations, the expressions of osteogenesis-related genes were higher in the BCP group than in the other groups. Meanwhile, to examine the osteogenic differentiation ability of intrascaffold cultured BMSCs, indicators were detected to assess the osteoinductivity of Ca-P bioceramics [31]. As shown in Fig. 4B, *Ocn*, *Bsp*, *Col1a1*, and *Runx2* expressions were augmented when BMSCs were cultured in BCP and β-TCP. Relative mRNA levels of *Ocn*, *Bsp*, *Col1a1*, and *Runx2* were significantly higher in the TCP group than in the other groups at all observation time points, which demonstrated that TCP is beneficial to osteogenesis. On the whole, the osteoconductivity of Ca-P bioceramics ranked as BCP > β-TCP > HA in vitro, whereas the osteoinductivity ranked as β-TCP > BCP > HA.

### In vivo osteoconductivity ranked as BCP > β-TCP ≥ HA, and osteoinductivity ranked as β-TCP ≥ BCP > HA

**Fig. 5:**
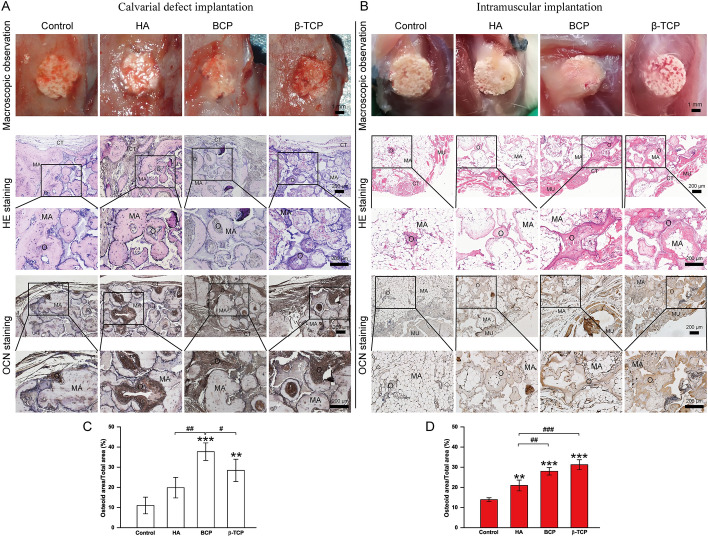
Comparative study of macroscopic observation and histological analysis in control, HA, BCP, and β-TCP groups in vivo. **A**, **B** Macroscopic observation, Hematoxylin–Eosin (HE) staining, and osteocalcin (Ocn) immunohistochemical staining of bioceramics implanted in calvarial defects (**A**) and muscles (**B**) at month 3 in control, HA, BCP, and β-TCP groups. **C**, **D** Osteoid area/total area of bioceramics implanted in calvarial defects and muscles at month 3 in control, HA, BCP, and β-TCP groups. CT = connective tissue, MA = material, MU = muscle, O = osteoid tissue

To verify the osteoconductivity and osteoinductivity of porous Ca-P scaffolds, Ca-P bioceramics were implanted in calvarial defects and muscles of rats. After 3 months of calvarial defect implantation and intramuscular implantation, skulls and muscles embedded with bioceramics were extracted and observed macroscopically and microscopically. As shown in Fig. 5A and B, neither abscess nor inflammation were found in the four groups, which revealed that infection control was effective and that these scaffolds had excellent histocompatibility. Scaffolds were in close contact with the native bone, and new bony tissues were found in the voids of bioceramics. More osteoids were found in the BCP and β-TCP groups than in the control and HA groups in both calvarial defect implantation and intramuscular implantation. Immunohistochemical staining of osteocalcin (OCN), a prominent marker of osteogenic differentiation, showed that positive staining was stronger in the HA, BCP, and β-TCP groups than in the control group. To evaluate the amount of newly formed bone in different groups, the ratio of osteoid area/total area was calculated based on immunohistochemical staining analysis with artificial intelligence image analysis software. As shown in Fig. 5C, the osteoid-specific area was significantly higher in the BCP group than in the other groups. However, in Fig. 5D, the percentage of osteoid formation was significantly higher in the β-TCP group than in the other groups, which demonstrated that osteoconductivity is not positively correlated with osteoinductivity in vivo.

### Differences in osteoconductivity and osteoinductivity of CaP bioceramics are associated with different Ca/P releasing ratios

**Fig. 6:**
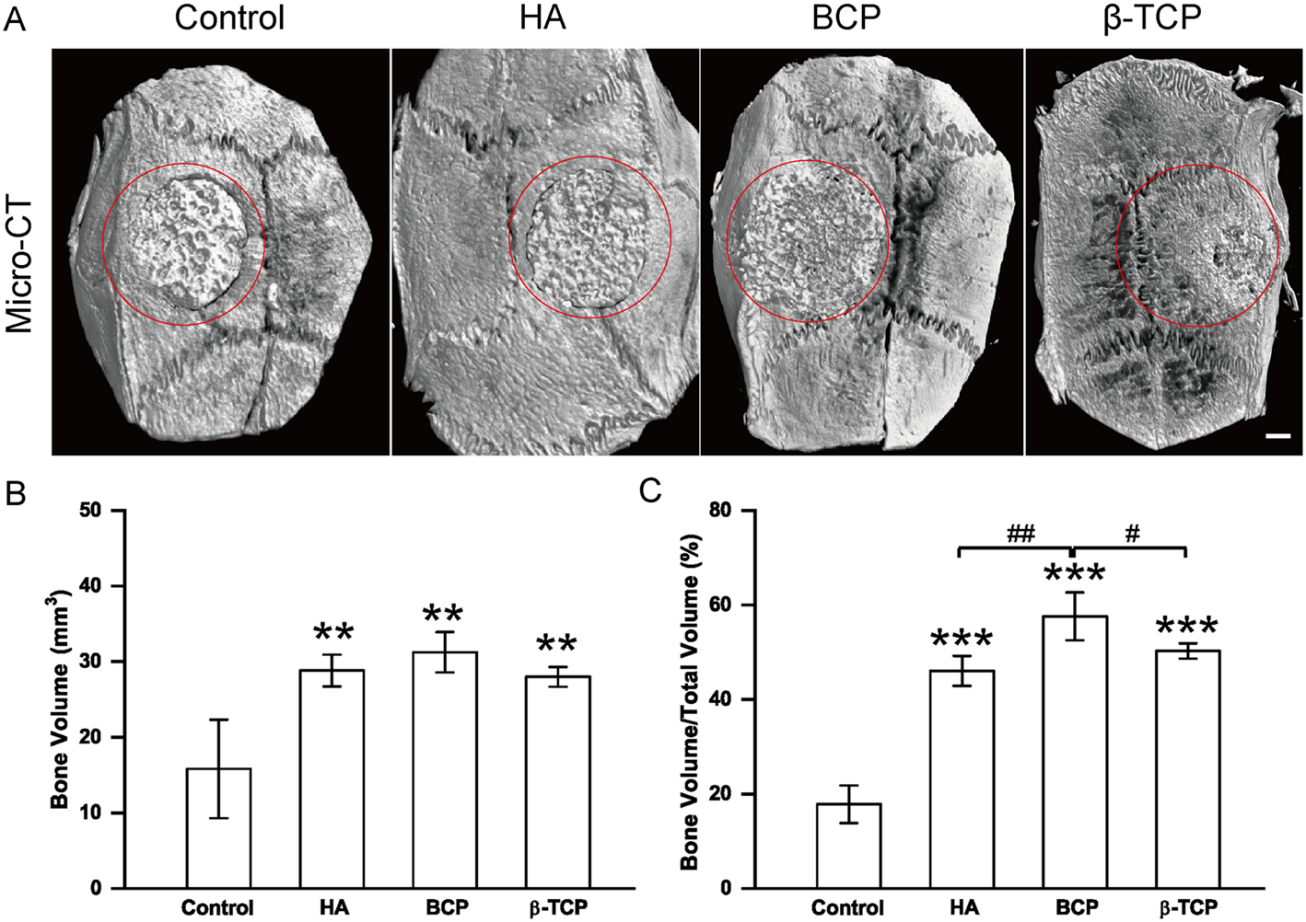
Image-based analysis of bioceramics implanted in calvarial defects at month 3 in the control, HA, BCP, and β-TCP groups. **A** Micro-computed tomography (micro-CT) of bioceramics (indicated with red circle) implanted in calvarial defects at month 3 in the control, HA, BCP, and β-TCP groups. **B**, **C** Bone volume and bone volume/total volume of bioceramics implanted in calvarial defects at month 3 in the control, HA, BCP, and β-TCP groups. All data points are presented as the mean ± standard deviation (*n* = 3). *, # indicates *P *< 0.05; **, ## indicates *P* < 0.01; ***, ### indicates *P* < 0.001. Scale bar = 1 mm

**Fig. 7:**
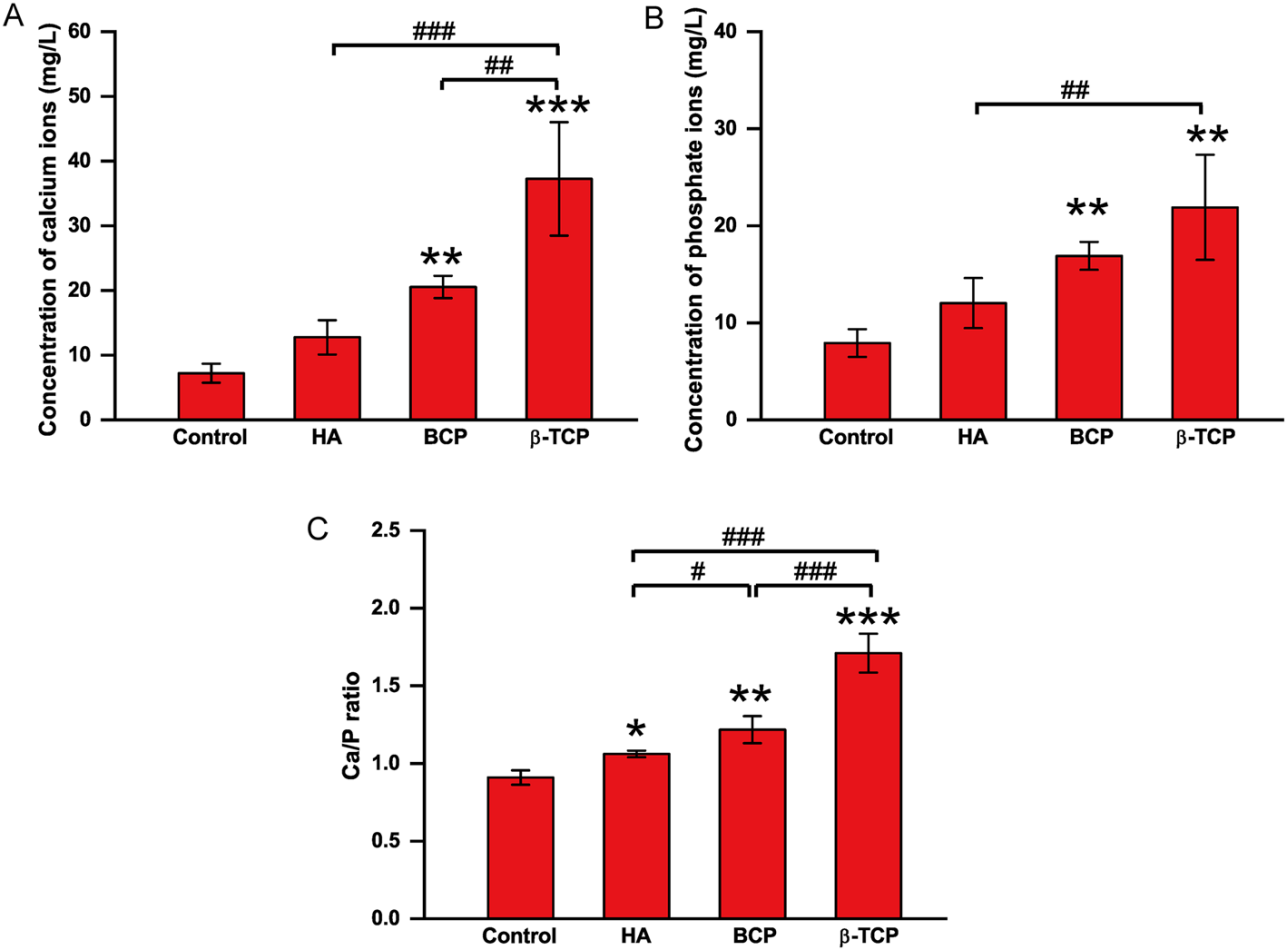
Concentrations of calcium and phosphate ions and Ca/P releasing ratio of bioceramic immersed solution in control, HA, BCP, and β-TCP groups on day 7. **A**,** B** Calcium and phosphate concentration of solution immersing four different bioceramics in control, HA, BCP, and β-TCP groups on day 7. **C** Ca/P ratio of solution immersing four different bioceramics in control, HA, BCP, and β-TCP groups on day 7. All data points are presented as the mean ± standard deviation (*n* = 3). *, # indicates *P* < 0.05; **, ## indicates *P* < 0.01; ***,### indicates *P* < 0.001

To investigate the factors that separate osteoconductivity and osteoinductivity, scaffold characteristics and the surrounding microenvironment were assessed with Micro-CT and ICP-OES. Micro-CT images were used to observe the integration between different Ca-P scaffolds and native tissues (Fig. 6A). When compared with the control and HA, BCP and β-TCP combined more closely with the native bone. Quantitative assay of bone volume and bone volume/total volume is shown in Fig. 6B and C. Bone volume was obviously higher in the HA, BCP, and β-TCP groups than in the control group, but there were no significant differences among the HA, BCP, and β-TCP groups. The bone volume/total volume ranked as BCP > β-TCP ≥ HA (Fig. 6C), and the bone volume/total volume ratio was strongly correlated with osteoconductivity. Owing to the environmental influence of osteogenic induction fluid, stem cells may be more susceptible to the local environment. Considering that all bioceramics had a similar interconnected pore structure with a porosity of 75 ± 5%, the ionic environment might be a vital influencing factor of osteoinductivity. As shown in Fig. 7A and B, the concentrations of calcium and phosphate ions in the β-TCP and BCP groups were significantly higher than those in the control and HA groups. Consistent with the trend of osteoinductivity, the trend of the Ca/P ratio was β-TCP (1.710 ± 0.125) > BCP (1.217 ± 0.087) > HA (1.061 ± 0.021) > control (0.909 ± 0.046) (Fig. 7C).

## Discussion

Osteoconductivity bridges the growth of bone to foreign materials [5]. In our study, BCP exerted a robust effect on osteoblasto-phenotypic characteristics in vitro and healing of calvarial defects in vivo, which is consistent with the previous studies [9, 10, 32]. As a composite of HA and β-TCP, BCP has the advantages of the two materials, and it is more beneficial to vascularization and integration while maintaining relatively slow degradation [33]. Biphasic bone substitute material (BSM, 60% HA and 40% β-TCP) has higher osteoconductivity and implant stability than monophasic BSM (100% β-TCP) in procedures of sinus floor augmentation [34]. A previous study showed that BCP induced less new bone formation but better volume preservation and more mature bone than β-TCP under constant pressure of cerebrospinal fluid [35]. These findings confirmed our study, although blood supply, ambient pressure, and animal species may cause the deviation between the performance of BCP and β-TCP. According to studies by Ebrahimi et al. on BCP, HA, and TCP (including α, α′, and β-TCP), the effect of physicochemical properties on clinical application is significant [36, 37]. Ensuring that the materials have similar microstructure is very important when studying the characteristics of each material, as the microarchitecture and biomaterial surface play important roles in regulating cell differentiation and macrophage responses to biomaterials [38, 39]. In our study, four bioceramics were fabricated with similar interconnected pore structures because scaffold characteristics including composition and phase transition are the main factors affecting osteoconductivity [40].

Osteoinductivity restricts osteoconductivity because it affects the differentiation of cells (especially stem cells or multipotent cells) during the process of bone regeneration [5]. A number of reports revealed that the osteoinductivity of BCP is higher than that of HA and β-TCP, and the strength of osteoinductivity increases with the content of β-TCP [12]. However, other studies demonstrated that the osteoinductivity of β-TCP is higher than that of BCP and HA [11, 41]. The contradictory results may be due to additional compounds used in the scaffolds and diverse physicochemical properties. Our study revealed that the osteoinductivity of β-TCP is stronger than that of BCP both in vitro and in vivo. Microenvironmental factors, such as dissolution or degradation of CaP-based biomaterials and local calcium ion concentration, may influence the results. The degradation of bioceramics in vitro is mainly affected by solubility, but in the in vivo environment, it is affected by a series of events, including the aggregation of macrophage immune responses, immunoregulation, and many other events [42]. Differences between the in vitro and in vivo environment may lead to different cellular responses [43–45]. Thus, investigating osteoconductivity and osteoinductivity both in vitro and in vivo is indispensable for revealing the association between osteoconductivity and osteoinductivity.

On the whole, osteoblastic phenotype in vitro and in situ as well as calvarial defect repair in vivo ranked as BCP > β-TCP > HA. Cell proliferation of pluripotent MSCs in vitro ranked as BCP > β-TCP > HA, and osteogenic differentiation markers of MSCs and heterotopic ossification in muscles in vivo ranked as β-TCP > BCP > HA. Based on the in vitro and in vivo experiments, we concluded that ion concentration influences the osteoinductivity of Ca-P bioceramics, and scaffold characteristics influence the osteoconductivity of Ca-P bioceramics. Rapid ion release improves the osteoinductivity of bioceramics but simultaneously reduces the cell attachment points. BCP is composed of HA with a stable structure and β-TCP with strong ion-releasing ability, and these characteristics may be responsible for its superior osteoconductivity.

## Conclusion

In this research, the osteoconductivity and osteoinductivity of HA, BCP, and β-TCP with similar pore structures and different Ca/P releasing ratios were investigated. Since all the Ca-P bioceramics in this study, which were fabricated using a hydrogen peroxide forming method, presented a similar interconnected pore structure with a porosity of 75 ± 5%, the diverse osteoconductivity and osteoinductivity of HA, BCP, and β-TCP were considered to be related to the calcium-to-phosphorus releasing ratio. Specifically, the Ca/P releasing ratios of β-TCP, BCP, HA, and control ceramics were 1.710 ± 0.125, 1.217 ± 0.087, 1.061 ± 0.021, and 0.909 ± 0.046, respectively. BCP showed better osteoconductivity in terms of osteoblastic phenotype in vitro and calvarial defect repair capacity in vivo, while β-TCP exerted better osteoinductivity in terms of the osteogenic differentiation of MSCs in vitro and heterotopic ossification in muscles in vivo. Thus, we believe that Ca/P releasing ratios of Ca-P bioceramics ranging from 1.5 to 2.0 are better for osteoinductivity, whereas osteoconductivity only requires a Ca/P releasing ratio of 1.0–1.5. More importantly, the relationship between osteoconductivity and osteoconductivity has long been unclear, and our study may provide a research direction for the future. However, the limitation of our study is that we only fabricated BCP by composing HA and β-TCP using a hydrogen peroxide forming method. Advanced fabrication methods, such as 3D printing, are suggested to generate Ca-P bioceramics with controllable Ca/P releasing ratios. In addition, the tunable Ca/P ratios of the Ca-P bioceramics and related genomics and proteomics studies for the construction of Ca-P bioceramics in bone regeneration are also worth investigating.

## Supplementary Information

**Additional file 1: Table S1.** SM Hit Listing of control sample.

**Additional file 2: Table S2.** SM Hit Listing of HA sample.

**Additional file 3: Table S3.** SM Hit Listing of BCP sample.

**Additional file 4: Table S4.** SM Hit Listing of β-TCP sample.

## Data Availability

The datasets used and/or analyzed during the current study are available from the corresponding author on reasonable request.
